# Supplementary material for: Histone H2A‐H2B binding by Pol α in the eukaryotic replisome contributes to the maintenance of repressive chromatin
Source: EMBO J. 2018 Aug 13;37(19):e99021. doi: 10.15252/embj.201899021 (PMC6166128; doi:10.15252/embj.201899021)
Supplement: Supplementary file 1 — Appendix [file EMBJ-37-e99021-s001.pdf]

Appendix for Evrin et al, "Histone H2A-H2B binding by Pol α in the eukaryotic replisome contributes to the maintenance of repressive chromatin"
Table of Contents:

Appendix Figure Legends S1-S8

Appendix Figure 1

Appendix Figure 2

Appendix Figure 3

Appendix Figure 4

Appendix Figure 5

Appendix Figure 6

Appendix Figure 7

Appendix Figure 8

Appendix Table S1

Appendix Figure Legends

Appendix Figure S1

Tethering of Pol α to the eukaryotic replisome is largely but not completely dependent upon Ctf4 and the CIP-box of Pol1. Control cells (YSS10), *ctf4Δ* (YGDP975), *pol1-4A* cells with mutations in the CIP-box (YCE1073) and *pol1-6A* with mutations in the histone-binding motif (YCE1075) were synchronized in G1-phase at 30°C and then released into S-phase for 20 minutes. S-phase samples used to prepare cell extracts, from which the CMG helicase component Mcm4 was isolated by immunoprecipitation. The associated replisome components were monitored by immunoblotting. The asterisk denotes a non-specific band in the cell extract samples that is recognized by the anti-Ctf4 antibody.

Appendix Figure S2

Telomere length is not affected by mutation of the CIP-box or histone-binding motifs of Pol1. Genomic DNA was extracted from the indicated strains and digested with Xho1, before transfer to nitrocellulose membrane and hybridization with a radiolabelled probe corresponding to the Y' element of yeast telomeres. Telomere length is reduced in *tel1Δ* cells that lack the Tel1 checkpoint kinase, and increased in *pol1-F1463A* cells in which primase is displaced from the carboxyl terminus of Pol1. In contrast, telomere length is not affected by histone-binding mutations in Mcm2 (Mcm2-3A) or Pol1 (Pol1-2A2 and Pol1-6A), or by displacement of Pol1 from the replisome (Pol1-4A).

Appendix Figure S3

Salt sensitivity of the interaction of Pol1NT with chromatin-derived histone complexes. Cells expressing ProteinA-tagged Pol1NT (YCE39) were grown and processed as for the experiments in Figure 3, except that the cell extracts were prepared in the presence of the indicated concentrations of KOAc.

Appendix Figure S4

Pol1NT and FACT can bind simultaneously to the same chromatin-derived histone complexes. (**A**) Repeat of the experiment in Figure 3E. The cells expressed GAL-Pol1NT and also had a second copy of *SPT16* at the *leu2* locus (in addition to the endogenous *SPT16* locus), expressing MYC-tagged versions of either wild type Spt16 (YCE248) or Spt16 with a small deletion at the carboxyl terminus (YCE250) that abolishes interaction of FACT with H3-H4 tetramers. After arresting cells in G2-M phase, cell extracts were prepared and treated with DNase to release chromatin-derived histone complexes, before isolation of Pol1NT by immunoprecipitation. Pol1NT associated in vitro with chromatin-derived histone complexes from both strains, but only co-purified with full-length Spt16-9MYC, rather than Spt16-ΔCT-9MYC (note that endogenous Spt16 was also present in both extracts, but is not shown in the figure). (**B**) MYC-tagged Spt16 was isolated from the same extracts, confirming that full-length Spt16-9MYC was able to co-purify with chromatin-derived histone complexes, but Spt16-9MYC was not. It was not possible to monitor association of MYC-tagged Spt16 with Pol1NT in this case, as the ProteinA tag on Pol1NT associated with the antibodies used to isolate Spt16-9MYC. (**C**) Similar experiment to those described above, except that expression of GAL-Pol1NT was induced in G1-phase cells that were then released into S-phase. Samples were taken 20' after release into S-phase and used to prepare cell extracts. (**D**) Pol1NT

was isolated by immunoprecipitation, after digestion of DNA to release histone complexes from chromatin. As above, Pol1NT co-purified with full-length Spt16-9MYC but not with the histone-binding mutant Spt16-ΔNT-9MYC. Pol1NT purified from both extracts with chromatin-derived histone complexes (probably with FACT containing endogenous wild type Spt16) and with the replisome (via the interaction of Pol1NT with trimeric Ctf4 that also binds directly to the CMG helicase).

Appendix Figure S5

Mutation of conserved residues in Pol1NT block association with chromatin-derived histones from S-phase or G2-M phase cells. (**A**) The amino terminal tails of wild type budding yeast Pol1 (YCE39) or Pol1-6A (YCE226) were induced in cells arrested in G2-M phase (lower panels), or in G1-phase cells before release into S-phase (top panels). (**B**) Samples were taken after induction in G2-M phase cells, or 20' after release into S-phase, and then used to prepare cell extracts, from which Pol1NT was isolated after digestion of DNA to release histone complexes from chromatin. The indicated proteins were monitored by immunoblotting. (**C**) Summary of the ability of Pol1NT with the indicated mutations to interact with Mcm2 and FACT (IPs from extracts not treated with DNase), or with Ctf4 and histones (IPs from extracts treated with DNase – the histone complexes co-purifying with Pol1NT are also bound by Mcm2 and FACT).

Appendix Figure S6

Functional analysis of the Pol1-2A2 and Pol1-6A alleles. (**A**) Control (YCE482) and *pol1-6A-9MYC* (YCE483) cells were synchronized in G1-phase at 30°C and then released into S-phase. Samples were taken after 20' and used to prepare cell

extracts, from which Pol1-9MYC (or Pol1-6A-9MYC) were isolated by immunoprecipitation. The indicated proteins were monitored by immunoblotting. (**B**) DNA content was monitored by flow cytometry in the same experiment. (**C**) Control (YCE542) and *pol1-2A2* (YCE1050) cells were synchronized in G1-phase at 24°C and then released into S-phase for 30', before replisome assembly was monitored as in Figure 6A, via immunoprecipitation of the Sld5 subunit of the CMG helicase. (**D**) The kinetics of DNA synthesis for the *pol1-2A2* mutant were monitored by flow cytometry, in the same experiment shown above in Figure 1A and Figure 6B (alongside control, *pol1-4A* and *pol1-6A*). Therefore, the control sample in this panel is identical to that shown in Figure 1A and Figure 6B.

Appendix Figure S7

Cells lacking the Dpb3 and Dpb4 subunits of DNA polymerase ε are viable in the absence of the S-phase checkpoint pathway. Diploid cells of the indicated genotype were processed as for Figure 1C-D.

Appendix Figure S8

Amino acid sequences of recombinant tagged forms of HsPOLA1-NTD (1-110) and HsMCM2-NTD (43-160). The sequences of HsPOLA1-NTD and HsMCM2-NTD are in blue, the recognition site for PreScission protease is in orange, the Twin-Strep tag is shown in green and the residues mutated to Alanine in HsPOLA1-NTD are in bold and red.

Evrin et al, Appendix Figure S1

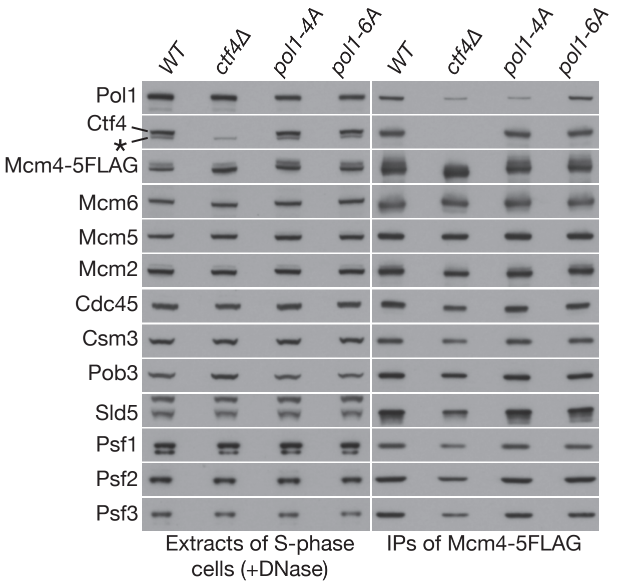

Evrin et al, Appendix Figure S2

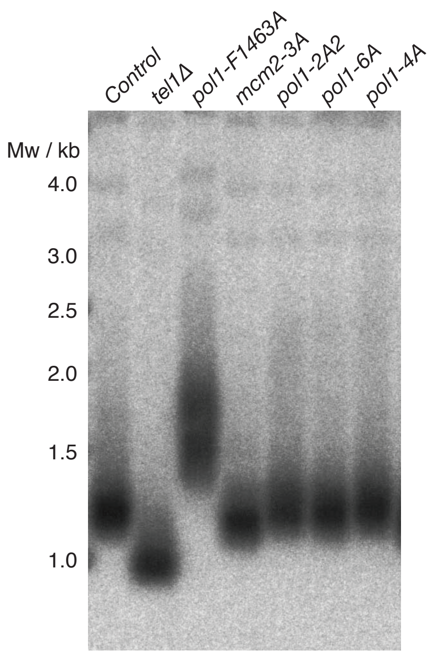

XhoI-digested genomic DNA
hybridised with probe to telomeric DNA

Evrin et al, Appendix Figure S3

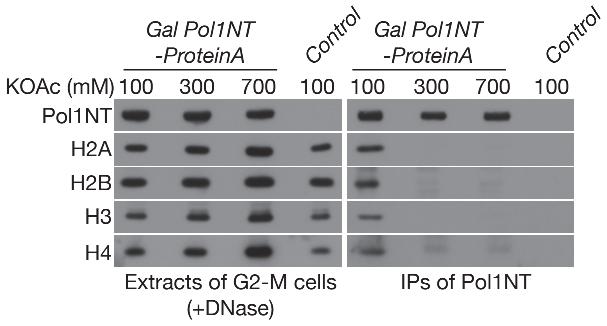

Evrin et al, Appendix Figure S4

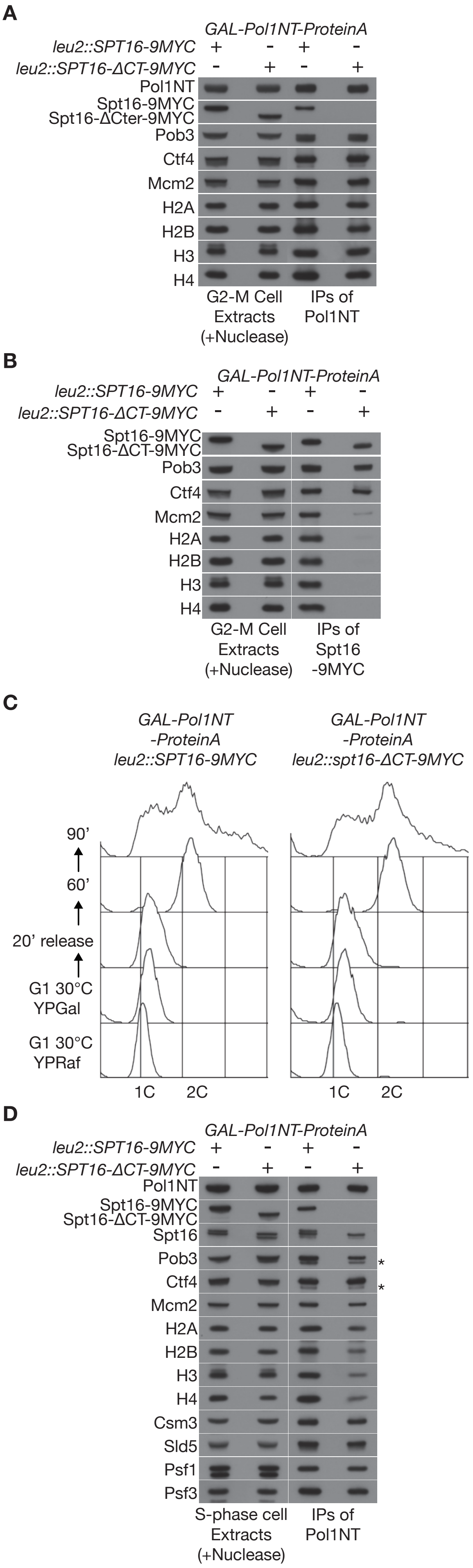

Evrin et al, Appendix Figure S5

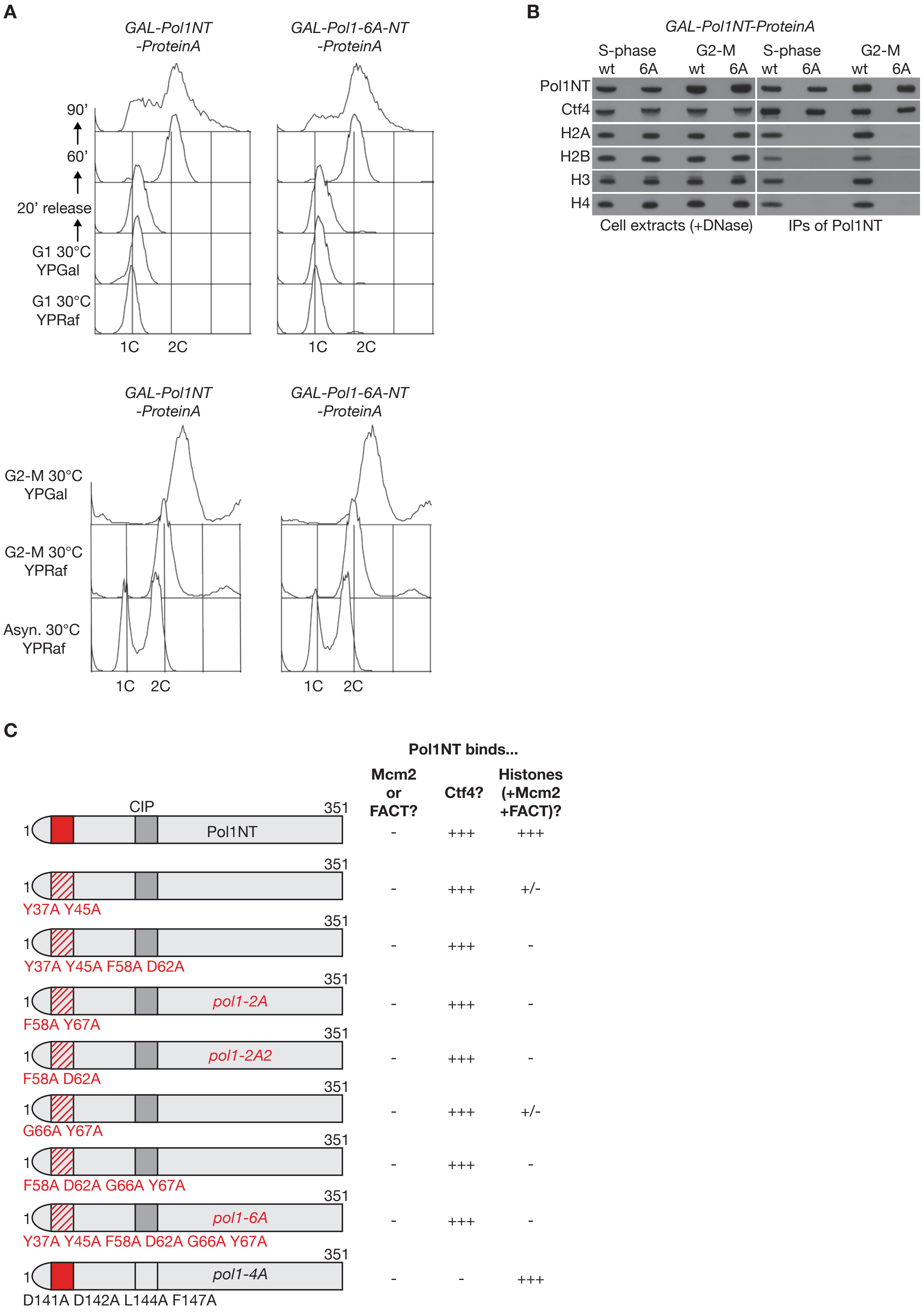

Evrin et al, Appendix Figure S6

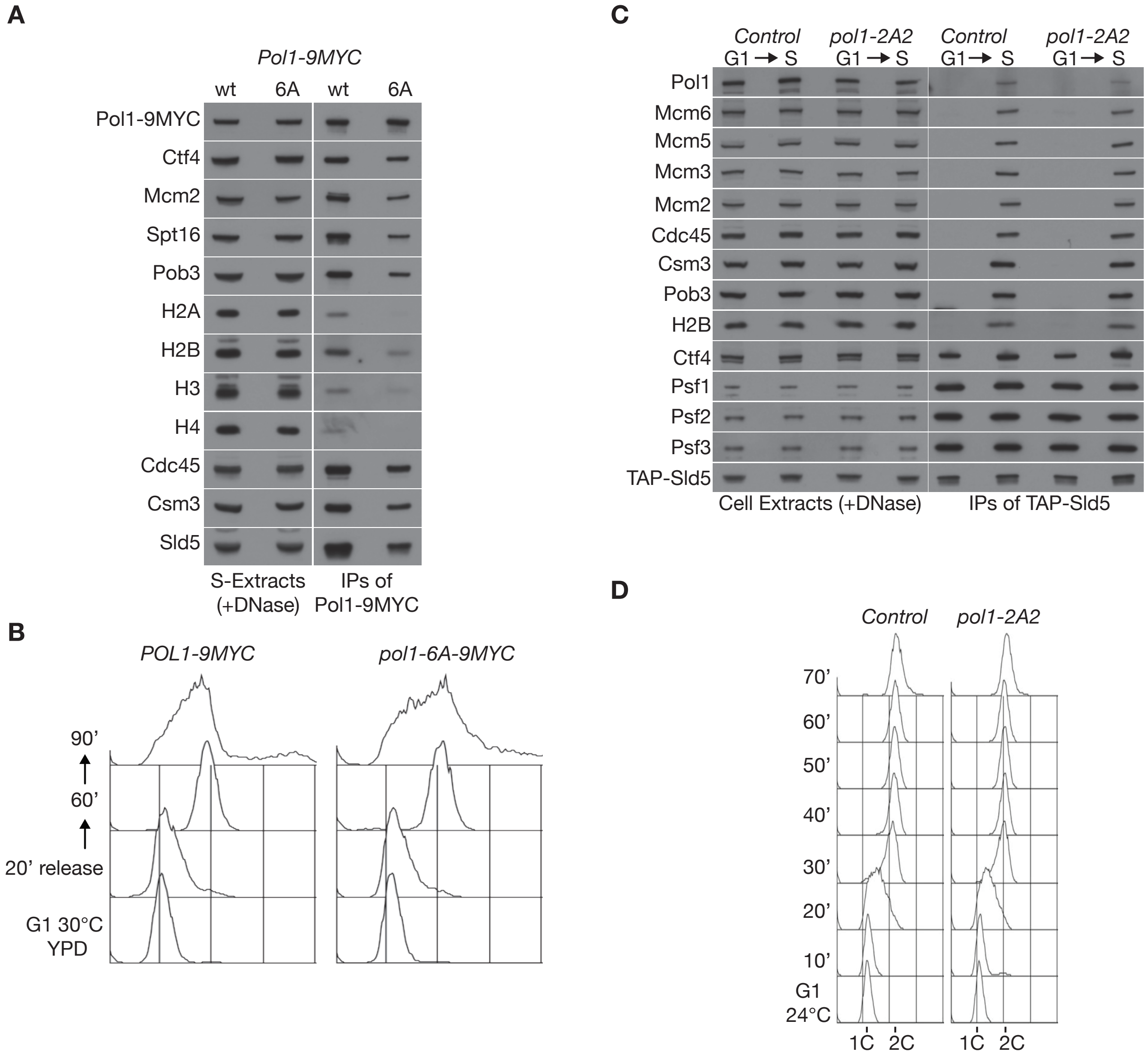

Evrin et al, Appendix Figure S7

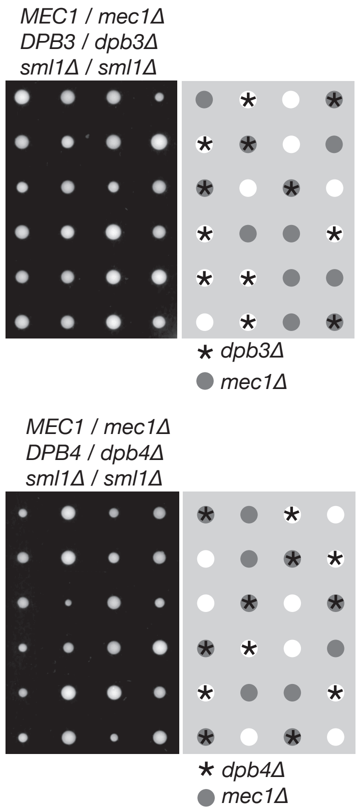

Evrin et al, Appendix Figure S8

LEVLFQGP = Precision Protease site

SAWSHPQFEKGGGSGGGSGGGSWSHPQFEK = TwinStreptag

Sequences of HsPOLA1 and HsMCM2 are indicated in BLUE

Location of **3A** mutations in HsPOLA1 are indicated in **bold red**

8His-MBP_Precision protease site_HsPOLA1 (1-110)_TwinStreptag

MNGSHHHHHHHHTSMKIEEGKLVIWINGDKGYNGLAEVGKKFEKDTGIKVT
VEHPDKLEEKFPQVAATGDGPDIIFWAHDRFGGYAQSGLLAEITPDKAFQDK
LYPFTWDAVRYNGKLIAYPIAVEALSLIYNKDLLPNPPKTWEEIPALDKELKAK
GKSALMFNLQEPYFTWPLIAADGGYAFKYENGKYDIKDVGVDNAGAKAGLT
FLVDLIKNKHMNADTDYSIAEAAFNKGETAMTINGPWAWSNIDTSKVNYGVT
VLPTFKGQPSKPFVGVLSAGIDAASPNKELAKEFLENYLLTDEGLEAVNKDK
PLGAVALKSYEEELAKDPRIAATMENAQKGEIMPNIPQMSAFWYAVRTAVIN
AASGRQTVDEALKDAQTNSSSTGSGTSGSTLEVLFQGPGSMAPVHGDDSL
SDSGSFVSSRARREKKSKKGRQEALERLKKAKAGEKYKYEVEDFTGVYEEV
DEEQYSKLVQARQDD**DW**IVDDDGIG**Y**VEDGREIFDDDLEDDALDADEKG
SAWSHPQFEKGGGSGGGSGGGSWSHPQFEK

8His-MBP_Precision protease site_HsPOLA1-3A (1-110)_TwinStreptag

MNGSHHHHHHHHTSMKIEEGKLVIWINGDKGYNGLAEVGKKFEKDTGIKVT
VEHPDKLEEKFPQVAATGDGPDIIFWAHDRFGGYAQSGLLAEITPDKAFQDK
LYPFTWDAVRYNGKLIAYPIAVEALSLIYNKDLLPNPPKTWEEIPALDKELKAK
GKSALMFNLQEPYFTWPLIAADGGYAFKYENGKYDIKDVGVDNAGAKAGLT
FLVDLIKNKHMNADTDYSIAEAAFNKGETAMTINGPWAWSNIDTSKVNYGVT
VLPTFKGQPSKPFVGVLSAGIDAASPNKELAKEFLENYLLTDEGLEAVNKDK
PLGAVALKSYEEELAKDPRIAATMENAQKGEIMPNIPQMSAFWYAVRTAVIN
AASGRQTVDEALKDAQTNSSSTGSGTSGSTLEVLFQGPGSMAPVHGDDSL
SDSGSFVSSRARREKKSKKGRQEALERLKKAKAGEKYKYEVEDFTGVYEEV
DEEQYSKLVQARQDD**AA**IVDDDGIG**A**VEDGREIFDDDLEDDALDADEKG
SAWSHPQFEKGGGSGGGSGGGSWSHPQFEK

8His-MBP_Precision protease site_HsMCM2 (43-160)_TwinStreptag

MNGLNDIFEAQKIEWHESGSHHHHHHHHTSMKIEEGKLVIWINGDKGYNGL
AEVGKKFEKDTGIKVTVEHPDKLEEKFPQVAATGDGPDIIFWAHDRFGGYAQ
SGLLAEITPDKAFQDKLYPFTWDAVRYNGKLIAYPIAVEALSLIYNKDLLPNPP
KTWEEIPALDKELKAKGKSALMFNLQEPYFTWPLIAADGGYAFKYENGKYDI
KDVGVDNAGAKAGLTFLVDLIKNKHMNADTDYSIAEAAFNKGETAMTINGPW
AWSNIDTSKVNYGVTVLPTFKGQPSKPFVGVLSAGIDAASPNKELAKEFLEN
YLLTDEGLEAVNKDKPLGAVALKSYEEELAKDPRIAATMENAQKGEIMPNIP
QMSAFWYAVRTAVINAASGRQTVDEALKDAQTNSSSTGSGTSGSTLEVLFQGP
GSGRDLPPFEDESEGLLGTEGPLEEEEDGEELIGDGMERDYRAIPELDAYEAE
GLALDDEDVEELTASQREAAERAMRQRDREAGRGLGRMRRGLLYDSDEEDE
ERPARKRRQVERATEDENLYFQGSAWSHPQFEKGGGSGGGSGGGSWSHPQFEKGSS

Strain	Genotype
W303-1	*MAT***a** *ade2-1 ura3-1 his3-11,15 trp1-1 leu2-3,112 can1-100 /*<br>*MAT*α *ade2-1 ura3-1 his3-11,15 trp1-1 leu2-3,112 can1-100*
W303-1a	*MAT***a** *ade2-1 ura3-1 his3-11,15 trp1-1 leu2-3,112 can1-100*
YSS3	*MAT***a** *pep4Δ::ADE2*
YGDP5	*MAT***a** *mec1Δ::ADE2 sml1Δ::HIS3*
YSS10	*MAT***a** *MCM4-5FLAG-9HIS (hphNT) pep4Δ::ADE2*
YFV18	*MAT*α *dna2-4A-9MYC*
YFV37	*MAT***a** *leu2::mURA3-LEU2*
YFV38	*MAT***a** *RDN1-NTS2::mURA3-LEU2*
YCE39	*MAT***a** *ura3-1::GAL-Pol1 1-351-ProteinA-7His (URA3) pep4Δ::ADE2*
YFV39	*MAT***a** *RDN1-NTS1::mURA3-LEU2*
YCE125	*MAT***a** *ura3-1::GAL-Pol1 1-200-ProteinA-7His (URA3) pep4Δ::ADE2*
YCE126	*MAT***a** *ura3-1::GAL-Pol1 1-267-ProteinA-7His (URA3) pep4Δ::ADE2*
YCE127	*MAT***a** *ura3-1::GAL-Pol1 1-315-ProteinA-7His (URA3) pep4Δ::ADE2*
YCE128	*MAT***a** *ura3-1::GAL-Pol1 89-351-ProteinA-7His (URA3) pep4Δ::ADE2*
YCE129	*MAT***a** *ura3-1::GAL-Pol1 150-351-ProteinA-7His (URA3) pep4Δ::ADE2*
YMP154-1	*MAT***a** *MCM2-9MYC (K.l.TRP1) pep4Δ::ADE2*
YMP177-1	*MAT***a** *SPT16-9MYC (K.l.TRP1) pep4Δ::ADE2*
YBH213	*MAT***a** *mrc1Δ::K.l.TRP1*
YCE217	*MAT***a** *ura3-1::GAL-Pol1-4A 1-351-ProteinA-7His (URA3) pep4Δ::ADE2*
YCE222	*MAT***a** *ura3-1::GAL-Pol1 G66A Y67A 1-351-ProteinA-7His (URA3) pep4Δ::ADE2*
YCE224	*MAT***a** *ura3-1::GAL-Pol1-F58A D62A G66A Y67A 1-351-ProteinA-7His (URA3) pep4Δ::ADE2*
YCE226	*MAT***a** *ura3-1::GAL-Pol1-6A 1-351-ProteinA-7His (URA3) pep4Δ::ADE2*
YCE232	*MAT***a** *ura3-1::GAL-Pol1-2A2 1-351-ProteinA-7His (URA3) pep4Δ::ADE2*
YCE234	*MAT***a** *ura3-1:: GAL-Pol1-Y37A Y45A F58A D62A 1-351 ProteinA-7His (URA3) pep4Δ::ADE2*
YCE236	*MAT***a** *ura3-1::GAL-Pol1-Y37A Y45A 1-351 ProteinA-7His (URA3) pep4Δ::ADE2*
YCE238	*MAT***a** *ura3-1::GAL-Pol1 1-351-ProteinA-7His (URA3) mcm2-3A (hphNT) pep4Δ::ADE2*
YCE248	*MAT***a** *ura3-1::GAL-Pol1 1-351-ProteinA-7His (URA3) leu2-3,112::SPT16-9MYC (LEU2) pep4Δ::ADE2*
YCE250	*MAT***a** *ura3-1::GAL-Pol1 1-351-ProteinA-7His (URA3) leu2-3,112::Spt16 1-957-9MYC (LEU2) pep4Δ::ADE2*

YCE278	*MAT***a** *pol1-6A-9MYC (hphNT)*
YMP287	*MAT***a** *ura3-1::GAL-Mcm2 1-200-ProteinA-7His (URA3) pep4Δ::ADE2*
YMP289	*MAT***a** *ura3-1::GAL-Mcm4 1-186 -ProteinA-7His (URA3) pep4Δ::ADE2*
YCE296	*MAT***a** *mcm2-3A-9MYC (K.l.TRP1) pol1-6A-9MYC (hphNT)*
YCE378	*MAT***a** *ura3-1::GAL-Sp Pol1 1-328-ProteinA-7His (URA3) pep4Δ::ADE2*
YCE405	*MAT***a** / *MATα VR/VR::ADE2-TEL VIIL/VIIL-adh4::URA3-TEL POL1/pol1-6A-9MYC (hphNT)*
YCE407	*MAT***a** *ura3-1::GAL-Hs Pol1 1-339-ProteinA-7His (URA3) pep4Δ::ADE2*
YLS409	*MATα hmrΔ77-144:SUP4-o HMR::ADE2* (*Mol. Cell. Biol.* **13**, 3919-3928, (1993))
YLS410	*MATα hmrΔ77-144:SUP4-o HMR::2EDA* (*Mol. Cell. Biol.* **13**, 3919-3928, (1993))
YCE425	*MAT***a** *ura3-1::GAL-Hs Pol1 1-339-2A2-ProteinA-7His (URA3) pep4Δ::ADE2*
YCE435	*MAT***a** *ura3-1::GAL-Hs Pol1 1-339-6A-ProteinA-7His (URA3) pep4Δ::ADE2*
YCE449	*MAT***a** *pol1-4A (URA3)*
YCE451	*MAT***a** *pol1-6A (URA3)*
YCE482	*MAT***a** *POL1-9MYC (HphNT) pep4Δ::ADE2*
YCE483	*MAT***a** *pol1-6A-9MYC (HphNT) pep4Δ::ADE2*
YCE485	*MAT***a** / *MATα VR/VR::ADE2-TEL*
YCE488	*MAT***a** / *MATα VR/VR::ADE2-TEL POL1/pol1-4A (URA3)*
YCE486	*MAT***a** / *MATα VR/VR::ADE2-TEL MCM2/mcm2-3A (hphNT)*
YMP502-5	*MAT***a** *mcm2-3A-9MYC (K.l.TRP1)*
YMP531	*MAT***a** *mcm2-3A (hphNT)*
YCE533	*MAT***a** / *MATα MEC1/mec1Δ::ADE2 sml1Δ::HIS3/sml1Δ::HIS3 POL1 / pol1-4A (URA3)*
YCE534	*MAT***a** / *MATα MEC1/mec1Δ::ADE2 sml1Δ::HIS3/sml1Δ::HIS3 POL1 / pol1 F1463A (hphNT)*
YCE535	*MAT***a** / *MATα MEC1/mec1Δ::ADE2 sml1Δ::HIS3/sml1Δ::HIS3 POL1 / pol1-6A (URA3)*
YCE542	*MAT***a** *TAP-SLD5 (kanMX) POL1 (URA3) pep4Δ::ADE2*
YCE544	*MAT***a** *TAP-SLD5 (kanMX) pol1-4A (URA3) pep4Δ::ADE2*
YCE546	*MAT***a** *TAP-SLD5 (kanMX) pol1-6A (URA3) pep4Δ::ADE2*
YCE555	*MAT***a** *pol1-2A2 (URA3)*
YCE597	*MAT***a** / *MATα MEC1 / mec1Δ::ADE2 sml1Δ::HIS3 / sml1Δ::HIS3 POL1 / pol1-2A (URA3)*
YCE609	*MAT***a** / *MATα VR/VR::ADE2-TEL POL1 / pol1-2A2 (URA3)*
YCE611	*MAT***a** / *MATα VR/VR::ADE2-TEL VIIL / VIIL::adh4-URA3-TEL TEL1 / tel1Δ::hphNT*
YCE641	*MAT***a** *leu2::mURA3-LEU2 mcm2-3A (hphNT)*
YCE643	*MAT***a** *leu2::mURA3-LEU2 pol1-2A2-9MYC (hphNT)*

YCE645	*MAT***a** *leu2::mURA3-LEU2 pol1-6A-9MYC (hphNT)*
YCE647	*MAT***a** *leu2::mURA3-LEU2 pol1-4A-9MYC (hphNT)*
YCE649	*MAT***a** *RDN1-NTS2::mURA3-LEU2 mcm2-3A (hphNT)*
YCE651	*MAT***a** *RDN1-NTS2::mURA3-LEU2 pol1-2A2-9MYC (hphNT)*
YCE653	*MAT***a** *RDN1-NTS2::mURA3-LEU2 pol1-6A-9MYC (hphNT)*
YCE655	*MAT***a** *RDN1-NTS2::mURA3-LEU2 pol1-4A-9MYC (hphNT)*
YCE657	*MAT***a** *RDN1-NTS1::mURA3-LEU2 mcm2-3A (hphNT)*
YCE659	*MAT***a** *RDN1-NTS1::mURA3-LEU2 pol1-2A2-9MYC (hphNT)*
YCE661	*MAT***a** *RDN1-NTS1::mURA3-LEU2 pol1-6A-9MYC (hphNT)*
YCE663	*MAT***a** *RDN1-NTS1::mURA3-LEU2 pol1-4A-9MYC (hphNT)*
YCE665	*MAT***a** *RDN1-NTS1::mURA3-LEU2 mcm2-3A-9MYC (K.l.TRP1) pol1-6A-9MYC (hphNT)*
YCE667	*MAT***a** *RDN1-NTS2::mURA3-LEU2 mcm2-3A-9MYC (K.l.TRP1) pol1-6A-9MYC (hphNT)*
YCE669	*MAT***a** *leu2::mURA3-LEU2 mcm2-3A-9MYC (K.l.TRP1) pol1-6A-9MYC (hphNT)*
YCE674	*MAT***a** *leu2::mURA3-LEU2 top1Δ::kanMX*
YCE676	*MAT***a** *RDN1-NTS2::mURA3-LEU2 top1Δ::kanMX*
YCE678	*MAT***a** *RDN1-NTS1::mURA3-LEU2 top1Δ::kanMX*
YCE694	*MAT***a** *leu2::mURA3-LEU2 dpb4Δ::kanMX*
YCE706	*MAT***a** *RDN1-NTS2::mURA3-LEU2 dpb4Δ::kanMX*
YCE718	*MAT***a** *RDN1-NTS1::mURA3-LEU2 dpb4Δ::kanMX*
YCE728	*MAT***a** *leu2::mURA3-LEU2 dpb3Δ::kanMX*
YCE735	*MAT***a** *RDN1-NTS2::mURA3-LEU2 dpb3Δ::kanMX*
YCE740	*MAT***a** *RDN1-NTS1::mURA3-LEU2 dpb3Δ::kanMX*
YCE797	*MAT***a** */ MATα MEC1/mec1Δ::ADE2 sml1Δ::HIS3 / sml1Δ::HIS3 DPB3 / dpb3Δ::kanMX*
YCE798	*MAT***a** */ MATα MEC1/mec1Δ::ADE2 sml1Δ::HIS3 / sml1Δ::HIS3 DPB4 / dpb4Δ::kanMX*
YCE799	*MAT***a** */ MATα VR/VR::ADE2-TEL TOF2 / tof2-4A*
YGDP975	*MAT***a** *MCM4-5FLAG-9HIS (HphNT) ctf4 Δ::kanMX pep4Δ::ADE2*
YCE1027	*MAT***a** *ura3-1::GAL-Pol1 1-351-ProteinA-7His (URA3) tel1Δ::hphNT pep4Δ::ADE2*
YGDP1045	*MAT***a** *tel1Δ::hphNT*
YCE1050	*MAT***a** *TAP-SLD5 (kanMX) pol1-2A2 (URA3) pep4Δ::ADE2*
YCE1073	*MAT***a** *MCM4-5FLAG-9HIS (hphNT) pol1-4A (URA3) pep4Δ::ADE2*
YCE1075	*MAT***a** *MCM4-5FLAG-9HIS (hphNT) pol1-6A (URA3) pep4Δ::ADE2*
YCE1084	*MAT***a** *ura3-1::GAL-Pol1-2A 1-351-ProteinA-7His (URA3) pep4Δ::ADE2*
YCE1120	*MAT***a** */ MATα HMR / HMR::ADE2*

YCE1121	*MAT***a** / *MATα HMR/ HMR::ADE2 MCM2 / mcm2-3A (HphNT)*
YCE1123	*MAT***a** / *MATα HMR / HMR::ADE2 POL1 / pol1-4A (URA3)*
YCE1124	*MAT***a** / *MATα HMR / HMR::ADE2 POL1 / pol1-6A (URA3)*
YCE1125	*MAT***a** / *MATα HMR / HMR::ADE2 POL1 / pol1-2A2 (URA3)*
YCE1126	*MAT***a** / *MATα HMR / HMR::2EDA*
YCE1127	*MAT***a** / *MATα HMR / HMR::2EDA MCM2 / mcm2-3A (HphNT)*
YCE1129	*MAT***a** / *MATα HMR / HMR::2EDA POL1 / pol1-4A (URA3)*
YCE1130	*MAT***a** / *MATα HMR / HMR::2EDA POL1 / pol1-6A (URA3)*
YCE1131	*MAT***a** / *MATα HMR / HMR::2EDA POL1 / pol1-2A2 (URA3)*
YGDP1135	*MAT***a** *pol1 F1463A (hphNT)*

Appendix Table S1

Yeast strains used in this study. All strains are based on the W303 background.
