# Supplementary material for: Histone H2A‐H2B binding by Pol α in the eukaryotic replisome contributes to the maintenance of repressive chromatin
Source: EMBO J. 2018 Aug 13;37(19):e99021. doi: 10.15252/embj.201899021 (PMC6166128; doi:10.15252/embj.201899021)
Supplement: Supplementary file 2 — Expanded View Figures PDF [file EMBJ-37-e99021-s002.pdf]

Expanded View Figures

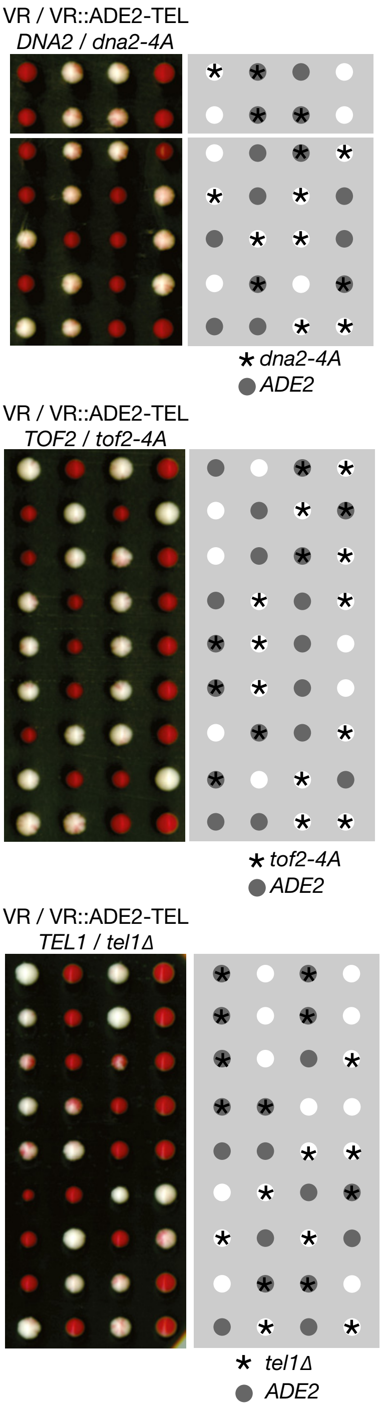

Figure EV1. Sub-telomeric silencing is not affected by CIP-box mutations in the Ctf4 partners Dna2 or Tof2, or by deletion of the *TEL1* gene.

The indicated diploids were processed as described above for Fig 2.

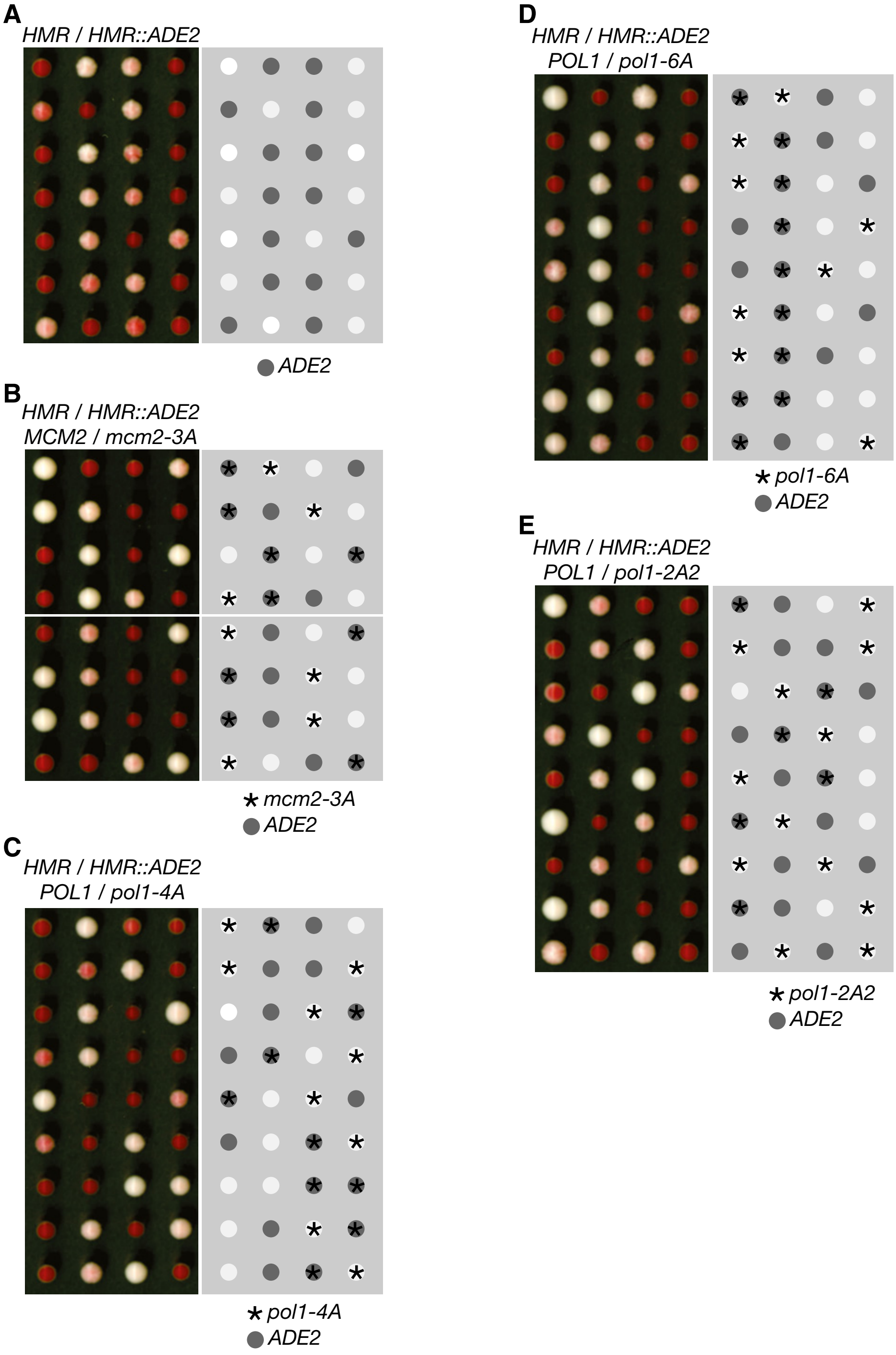

Figure EV2. Replisome-tethered histone-binding activity of Mcm2 and Pol alpha is required for gene silencing at the HMR mating-type locus.

A–E Tetrad analysis of meiotic progeny of the indicated diploids (heterozygous for insertion of the *ADE2* marker gene at the HMR locus on chromosome 3), processed as described in Materials and Methods. The promoter of the *ADE2* marker was positioned proximal to the *HMR-E* silencer element (denoted *HMR::ADE2*), and this corresponded to weaker silencing of *ADE2* compared to the *HMR::2EDA* strains in Fig 2D–F. We examined a total of 16 *mcm2-3A HMR::ADE2* colonies, 16 *pol1-4A HMR::ADE2* colonies, 15 *pol1-2A2 HMR::ADE2* colonies and 16 *pol1-6A HMR::ADE2* colonies.

© 2018 The Authors

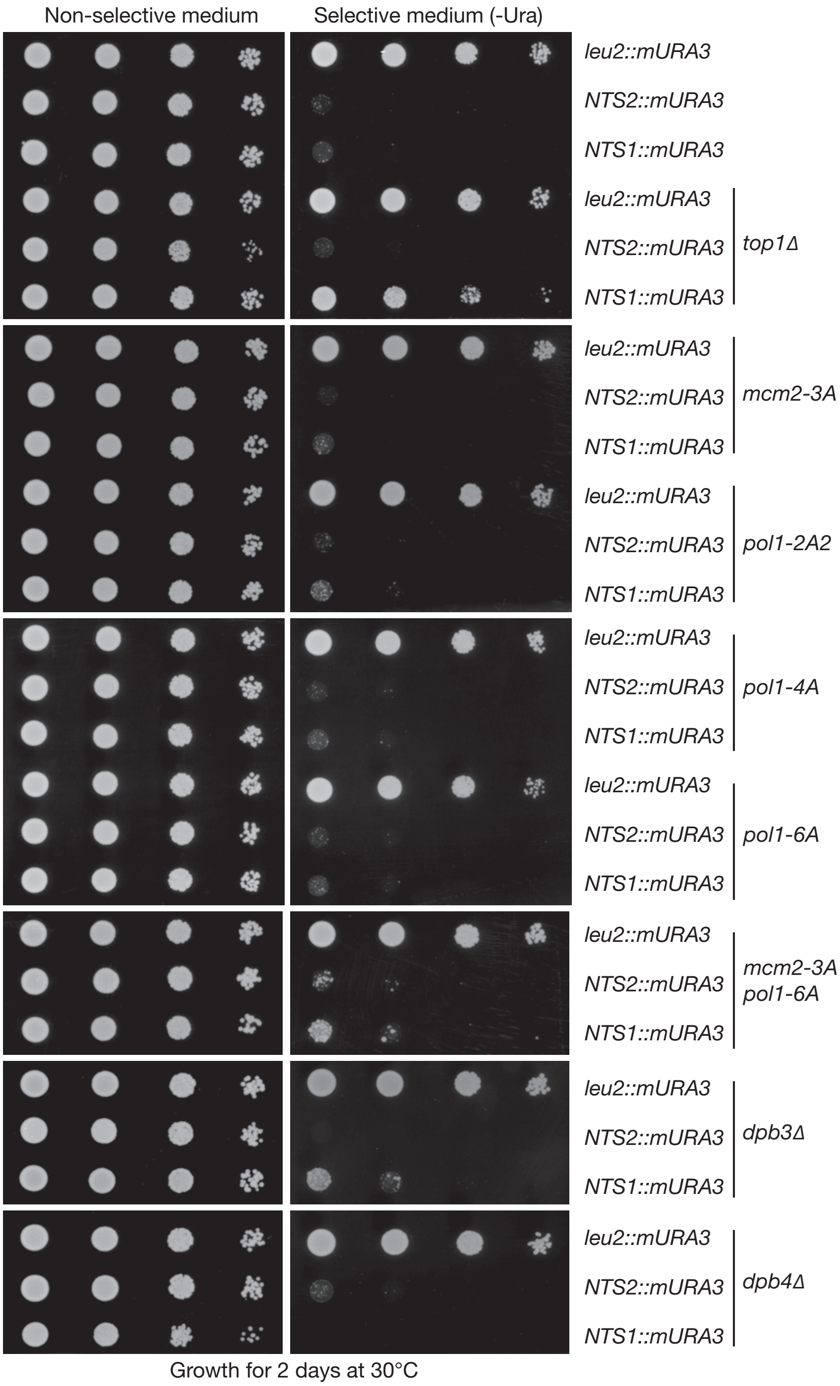

Figure EV3. Histone-binding activities of replisome proteins are largely dispensable for gene silencing within the rDNA repeats.

Serial dilutions of the indicated strains, in which the "modified *URA3* marker" (mURA3) was inserted at the *leu2* locus or else within the "Non-transcribed spacers" of an rDNA repeat (*NTS1* and *NTS2*), were grown on non-selective or selective medium, as described in Materials and Methods. In control cells, m*URA3* can be expressed from the *leu2* locus but is repressed when present at the *NTS1* or *NTS2* element of an rDNA repeat. Repression at *NTS1* was lost in *top1*Δ cells, as seen previously (Huang *et al*, 2006), but an equivalent defect was not observed in *mcm2-3A*, *pol1-2A2*, *pol1-4A*, *pol1-6A*, *mcm2-3A pol1-6A*, *dpb3*Δ or *dpb4*Δ.

© 2018 The Authors

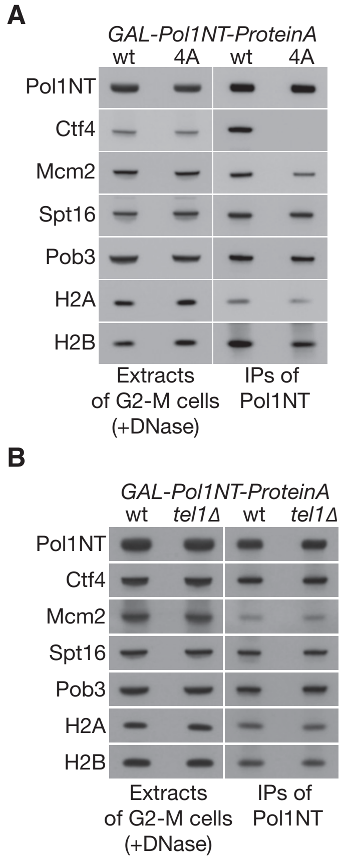

Figure EV4. Neither Ctf4 nor Tel1 is required for the histone-binding activity of Pol1NT.

A Mutation of the CIP-box of Pol1 displaces Ctf4 without affecting the interaction with histones released from chromatin.

B Although Tel1 is a novel partner of Pol1NT, deletion of the *TEL1* gene does not affect histone-binding by Pol1NT.

 © 2018 The Authors

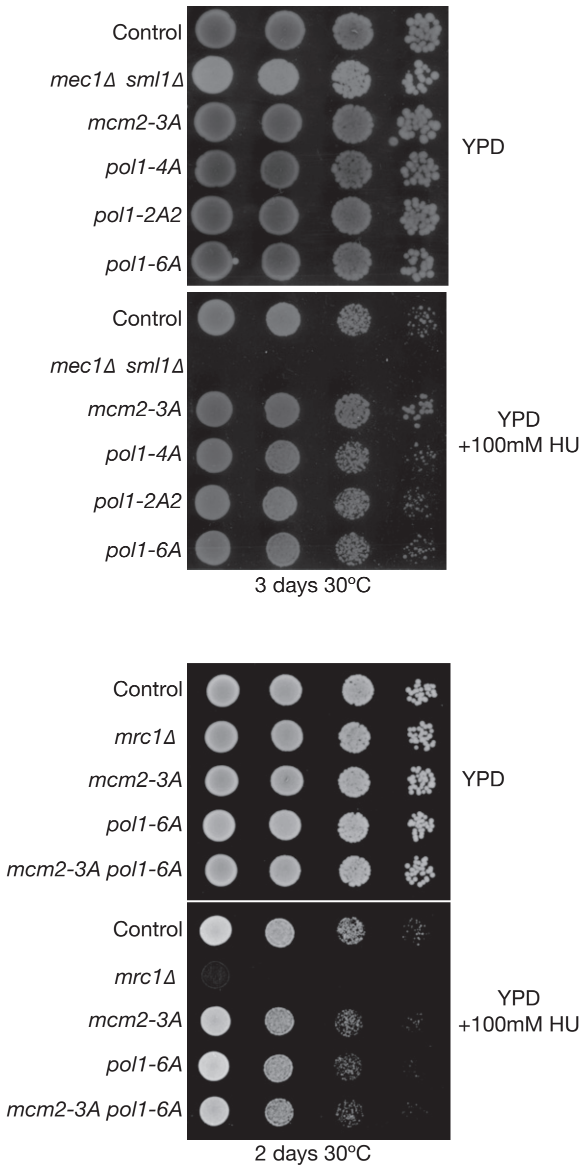

Figure EV5. Mutations in the histone-binding motifs of Mcm2 or Pol1 do not cause sensitivity to hydroxyurea treatment.

Serial dilutions of cells were grown on the indicated media for 2–3 days at 30°C before imaging.

© 2018 The Authors
